# Insertion outside the supersite in the N-terminal domain of the SARS-CoV-2 spike glycoprotein may contribute to evasion of neutralizing antibodies

**DOI:** 10.3389/fmicb.2026.1912073

**Published:** 2026-09-18

**Authors:** Elena A. Ermolaeva, Anastasia D. Fomina, Anna N. Zyrina

**Affiliations:** 1 Laboratory of Biochemistry, Chumakov FSC R&D IBP RAS/Institute of Poliomyelitis, Moscow, Russia; 2 Laboratory of Antivirals, Chumakov FSC R&D IBP RAS/Institute of Poliomyelitis, Moscow, Russia

**Keywords:** evasion of the immune response, glycoprotein spike, homology modeling, insertion, ntd, SARS-CoV-2

## Abstract

Variants of the SARS-CoV-2 virus continue to evolve. Rapidly emerging mutations, particularly in the Spike glycoprotein, may increase transmissibility and infectivity, and also lead to evasion of the immune response. The Spike protein contains two immunodominant epitopes: NTD and RBD. Numerous neutralizing antibodies against NTD are found in patient sera, and most of these target “supersite” epitopes. Consequently, mutations outside this region receive less attention. Our work continues the investigation of the effect of insertions outside the “supersite” on the phenotypic properties of SARS-CoV-2. Previously we characterized two closely related SARS-CoV-2 Delta variants differing by a 12-nucleotide insertion in the N-terminal domain of the Spike glycoprotein. In the present study, we modeled these two Spike glycoproteins and used them to perform spatial alignment analyses of S-protein structures in complex with antibodies against the NTD to assess the potential impact of the insertion on virus binding by neutralizing antibodies. We identified four potential scenarios, how the insertion can impact the binding of neutralizing antibodies targeting the NTD. Our study confirms the importance of investigating rapidly emerging mutations in the NTD, not only within the “supersite” but also beyond it. These mutations may contribute to immune evasion; consequently, their investigation is crucial for assessing the efficacy of commercially available monoclonal antibodies and vaccine preparations.

## 1 Introduction

Coronaviridae are a family of enveloped viruses with positive-sense RNA genome (V'kovski et al., 2021). One member of this family, SARS-CoV-2, causes the respiratory disease COVID-19, which can range from mild to fatal (Sasikumar and Unniappan, 2024). SARS-CoV-2 variants continue to evolve rapidly because of their mutation rate (Lauring and Andino, 2010). In particular, the rapid emergence of mutations in the Spike glycoprotein not only increases the transmissibility and infectivity of new variants, but also gives them an advantage in evading the host's immune response, whether developed as a result of infection or vaccination (Cui et al., 2022).

The Spike glycoprotein is the primary target of neutralizing antibodies, specifically its two domains, the receptor-binding domain (RBD, 319–541 residues) and the N-terminal domain (NTD, 14–305 residues). The RBD has been extensively studied because it mediates interaction with host cell's angiotensin-converting enzyme 2 (ACE2) receptor, which is essential for the viral entry. In contrast, considerably less attention has been paid to the NTD. However, interest in it has increased following the discovery of numerous neutralizing antibodies targeting NTD (Chi et al., 2020; Liu et al., 2020; Cerutti et al., 2021; McCallum et al., 2021).

The NTD of the Spike glycoprotein is divided into upper, central, and lower regions. The lower region is formed by a β-roll motif and an α-helix and connects NTD to the connector-part of S1 subunit (306–318, 542–685 residues) and thus to the RBD. The central structure consists of a galectin-like antiparallel β-sandwich fold formed by six- and seven-stranded β-sheets. The upper region consists of two antiparallel β-sheets connected by a short loop, whilst the lower region consists of two short β-sheets and helices (Zhang et al., 2021). The most immunogenic region of the NTD is known as the “supersite”. The loops that form this region are in a disordered state in most of the Spike glycoprotein structures studied (they are flexible) but upon binding to antibodies, some of them adopt a rigid β-sheet or α-helix structure (Cerutti et al., 2021; Figure 1). It has been demonstrated that mutations in the NTD affect not only immunogenicity but also viral fusion with the target cell, despite the fact that this domain does not bind directly to the ACE2. The NTD plays an important role in maintaining the trimeric conformation through chemical interactions with neighboring domains of the Spike glycoprotein, including the RBD (Li et al., 2025). This was also confirmed in a cryo-EM study: the researchers demonstrated that when NTD binds to sialoglycans, conformational changes occur in its structure, leading to the RBD transitioning to an ‘up' state, which results in fusion with the receptor. The authors of the article suggest that this mechanism applies to all coronaviruses (Pronker et al., 2023). There is also another mechanism by which the NTD indirectly influences RBD binding to the receptor; for example, certain human antibodies directed against the NTD facilitate binding to ACE2 and infection of the host cell (Lusiany et al., 2023).

**Figure 1:**
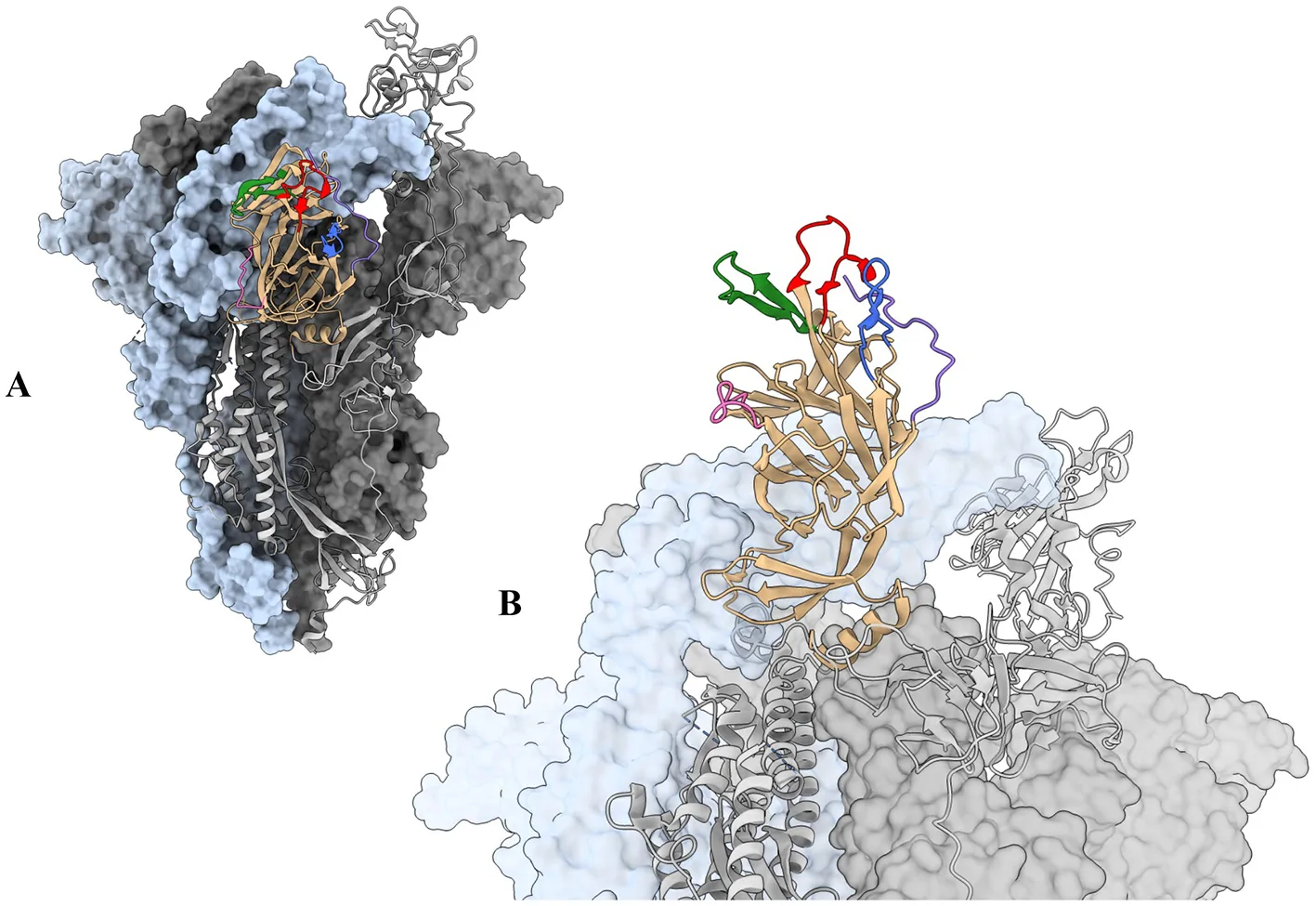
**(A)** Full structure of the Spike glycoprotein **(B)** Close-up view of the Spike glycoprotein NTD (highlighted in yellow). The “supersite” loops are marked: N1 (purple), N2 (blue), N3 (green), N4 (pink), and N5 (red). The RCSB PDB identifier for the SARS-CoV-2 Spike protein structure is 8HLD; the visualization was created using UCSF ChimeraX.

In some patients who have recovered from COVID-19, the levels of antibodies against the NTD in blood plasma, including neutralizing antibodies, may significantly exceed those against the RBD. Consequently, mutations in the NTD enable the virus to effectively evade the host immune response (Voss et al., 2021). For example, deletions in the “supersite” region result in the evasion of neutralizing antibodies, in particular the 4A8 antibody. Furthermore, the concurrent presence of a 140 deletion in the NTD and an E484K mutation in the RBD leads to a four-fold reduction in neutralization by polyclonal antibodies (Harvey et al., 2021), while a deletion of residues 242–244 can reduce the neutralizing activity of sera obtained from recovered patients by more than half (Wang et al., 2021). Therefore, it is necessary to study and characterize rapidly emerging mutations in the Spike glycoprotein, particularly in the region encoding the NTD, as they may influence immune evasion.

We had previously identified an uncharacterized insertion in the NTD, located outside the “supersite”, and we investigated its effect on plaque phenotype and antibody binding to the virus using an ELISA assay, as well as the effect on pathogenesis using the Syrian hamster as an animal model (Ermolaeva et al., 2026). This article forms part of a wider study assessing the impact of insertions outside the “supersite” on the properties of the Spike glycoprotein, specifically their effect on evading neutralizing antibodies produced by the host organism. To this end, a homology-based model of the spike glycoprotein was constructed, which contains the five-amino acid long insertion in the NTD outside the “supersite”. Using this model and complexes of the Spike glycoprotein with antibodies targeting the NTD obtained from open-access sources, spatial alignments were constructed to assess the potential impact of the insertion on antibody binding.

## 2 Materials and methods

### 2.1 Determination of the amino acid sequence of the spike glycoprotein

RNA was isolated from virus-containing fluid using an RNA extraction kit, followed by precipitation and treatment with DNase I. Fasta files containing the full genome were subjected to bioinformatic analysis (Ermolaeva et al., 2026). To obtain the sequence encoding the Spike glycoprotein, the full-genome sequences were aligned in MEGA-X 10.0.5 (Kumar et al., 2018) against the nucleotide sequence of the Wuhan-Hu-1 strain (NC_045512.2; severe acute respiratory syndrome coronavirus 2 isolate Wuhan-Hu-1) as a reference using the MUSCLE algorithm (Edgar, 2004). The conservation and uniqueness of the first and last 20 nucleotides of the sequences encoding the Spike glycoprotein were verified. The Spike glycoprotein- encoding regions were identified within the whole-genome sequences of Delta s.p. and Delta l.p variants and excised from the start codon to the stop codon. The resulting nucleotide sequences were translated into amino acid sequences using the EMBOSS package [transeq, 6.6.0.0., (Rice et al., 2000)]. The resulting amino acid sequences were validated by aligning them with the Spike glycoprotein of the Wuhan-Hu-1 strain (NCBI: YP_009724390.1) as a reference.

### 2.2 Search for Spike glycoprotein structures for homology modeling

A search was conducted in the PDB (Protein Data Bank; Berman, 2000) using the online Protein BLAST (Camacho et al., 2009) service to identify structures homologous to the amino acid sequence of the Delta variant's Spike glycoprotein, using the program's default settings and specifying the organism as “Viruses.” Alignment of the amino acid sequences of the selected structures (Delta s.p. and Delta l.p.) was performed with MEGA-X 10.0.5 program using the MUSCLE algorithm.

### 2.3 Homology modeling

Two Spike glycoprotein variants' structures have been modeled with the MODELER 10.5 program (Webb and Sali, 2016). Sequence alignments were reformatted manually for the generation of trimers (Supplementary Figure S1), first 13 residues of Delta s.p. and Delta l.p. were excluded to match experimental strictures. Three models were generated for each sequence with multiple templates, the best model being selected based on DOPE and GA341 functions (Supplementary Data 1, Supplementary Table S1, Supplementary Figures S3, S4). Models' quality was assessed with ProSa-web (Wiederstein and Sippl, 2007). Modeling was performed with implicit hydrogen atoms (AutoModel class). The “slow” optimization algorithm with a maximum number of iterations of 300 was used. Optimization was repeated twice. For the loop modeling four structures with different antibody-binding mode were chosen [7C2L (Chi et al., 2020; Cerutti et al., 2021), 7LQV (Cerutti et al., 2021), 7NTC (Rosa et al., 2021) and 8HLD (Zhang et al., 2024)]. The loop modeling for residues 99–103 of the NTD was executed with DOPELoopModel class with 1,000 models for each of the four reference structures individually with and without co-crystallized antibodies (Supplementary Data 2). Visualization was performed with UCSF ChimeraX program (Meng et al., 2023). For additional spatial alignment of the NTD region, the MatchMaker tool was used for amino acid residues 14–306. Buried area, RMSD, minimum distance between the insertion residues and antibody atoms and possible clashes between the models and antibodies were calculated with ChimeraX corresponding functions.

## 3 Results

We had previously obtained two phenotypically homogeneous SARS-CoV-2 Delta variants [Delta small plaque (s.p.) and Delta large plaque (l.p.)] from a phenotypically heterogeneous Delta isolate 4724d (GISAID EPI_ISL_8799478; Figure 2). A quantitative analysis (following a base-10 logarithmic transformation of plaque areas) showed that the difference in plaque size between the Delta s.p. and Delta l.p. variants was statistically significant (*p* < 0.0001).

**Figure 2:**
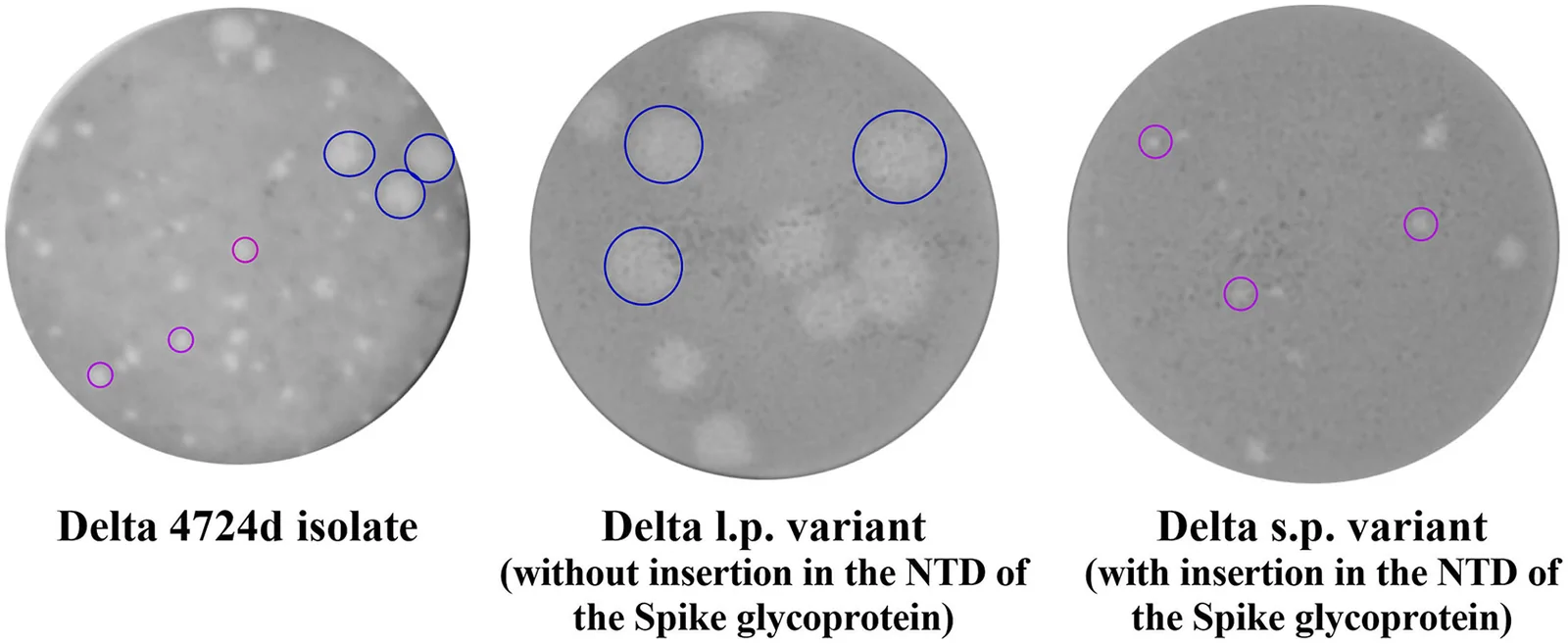
Plaque phenotypes of Delta strain 4729d, Delta l.p. variant (without insertion in the NTD of the Spike glycoprotein), and Delta s.p. variant (with insertion in the NTD of the Spike glycoprotein) under agar overlay in Vero cells on day 4 post infection.

The nucleotide sequences of the full-length genomes of the phenotypically homogeneous SARS-CoV-2 variants—Delta s.p. and Delta l.p.—were obtained using next-generation sequencing based on Illumina technology. The Delta s.p. variant contains an insertion resulting in the introduction of five new amino acids [N99K, Ins (RWRS)] in the NTD sequence of the Spike glycoprotein. This insertion is the only mutation distinguishing the Delta s.p. Spike glycoprotein from the Delta l.p. Spike glycoprotein (Supplementary Data 3). The variant with the insertion exhibited different antibody binding, as evidenced by a significantly lower optical density (OD) when both polyclonal and monoclonal antibodies were used in an ELISA experiment with the Delta s.p. variant as the antigen (the differences in OD when using the Delta s.p. and Delta l.p. variants as antigens were statistically significant, p < 0.0001). Based on the data obtained in the *in vitro* experiment, a hypothesis was put forward that the insertion we identified may influence evasion of neutralizing antibodies. In the present study, the S proteins of the two coronavirus variants were modeled to assess the possible influence of the insertion on the binding of neutralizing antibodies to the NTD (Ermolaeva et al., 2026).

### 3.1 Homology modeling of the Spike glycoprotein variants' structures

The nucleotide sequences encoding the Spike glycoprotein were obtained from the whole-genome nucleotide sequences of the SARS-CoV-2 Delta s.p. and Delta l.p. variants. Using the EMBOSS package (transeq, 6.6.0.0), the amino acid sequences of the S protein were derived from these. A search was conducted in the PDB for sequences homologous to the Spike glycoprotein sequence of Delta s.p. obtained, using the web version of the bioinformatics tool Protein BLAST, with the program's default settings.

80 SARS-CoV-2 Spike glycoprotein structures were selected based on the percentage of identical (PID) amino acids (89.6 %−99.8 %) with our sequence, as well as on the presence of a binding of the S protein and an antibody against the NTD in the PDB file. Alignments were then constructed in MEGA-X 10.0.5 using the MUSCLE algorithm (Supplementary Figure S1). Out of these 80 structures, 23 represented the complexes with NTD-targeting antibodies.

Selected structures were visually analyzed using ChimeraX 1.3, and regions with insufficient resolution in the NTD region were identified for each model. For further modeling, five structures were chosen that were representative of the conformational space of Spike glycoprotein complexes with antibodies targeting the NTD and possessed the fewest regions with insufficient resolution: 7UKL, 7C2L, 8HLD, 7L2C, 7L2D (PID 99.29 %−95.28 %; Table 1).

**Table 1:** PDB structures selected for the construction of the Spike glycoprotein based on homology.

PDB	Title	PID to Delta s.p. Spike glycoprotein	Unresolved sections in the NTD, aa	Antibody	Reference
7UKL	Cryo-EM structure of Antibody 12–16 in complex with prefusion SARS-CoV-2 Spike glycoprotein	99.29 %	69–80, 250–264, 143–159	12–16	Liu et al., 2023
7C2L	S protein of SARS-CoV-2 in complex bound with 4A8	99.21 %	250–257	4A8	Chi et al., 2020
8HLD	S protein of SARS-CoV-2 in complex with 26434	98.89 %	—	26434	Zhang et al., 2024
7L2C	Crystallographic structure of neutralizing antibody 2–51 in complex with SARS-CoV-2 spike N-terminal domain (NTD)	89.14 %	—	2–51	Cerutti et al., 2021
7L2D	Cryo-EM structure of NTD-directed neutralizing antibody 1–87 in complex with prefusion SARS-CoV-2 spike glycoprotein	94.86 %	—	1–87	Cerutti et al., 2021

Structural models of the spike glycoprotein for the Delta s.p. and Delta l.p. variants were constructed by homology modeling with the MODELER software, using the structures presented above as multiple templates. Models' quality was assessed with Ramachandran plots (Supplementary Figures S2, S3) and ProSA z-score (Supplementary Table S1). While z-scores for all models were in the favorable intervals for the corresponding protein length, many residues outside of the NTD were placed in disallowed regions of the Ramachandran plot. Nevertheless, NTD-only models contained no more than three residues, located in disallowed regions, and for the best models these residues were located far from the insertion loop. This allowed us to consider the models qualitative enough for further analysis. The resulting models were spatially aligned in ChimeraX using the MatchMaker tool with all 23 identified S protein- antibody complexes from the open-access PDB. Spatial alignment was performed over the amino acid sequence region from 14 aa to 305 aa to ensure the most accurate comparison of the NTD and antibodies' binding modes. These structures were further used for visual analysis, while for quantitative analysis four groups of models were built, for each of the representative antibody binding modes, to sample the conformational space of the insertion residues.

### 3.2 . Analysis of the spatial alignments of the Delta s.p. and Delta l.p. variants with S-protein structures in complex with antibodies against the NTD

Based on a visual analysis of the spatial alignment of the Delta s.p. variant of the S protein model with all selected structures of glycoprotein S complexes with antibodies, we identified four potential scenarios, in which the insertion might affect antibody binding. The structures 7C2L, 7LQV, 7NTC and 8HLD were selected for their visualization (Figure 3).

**Figure 3:**
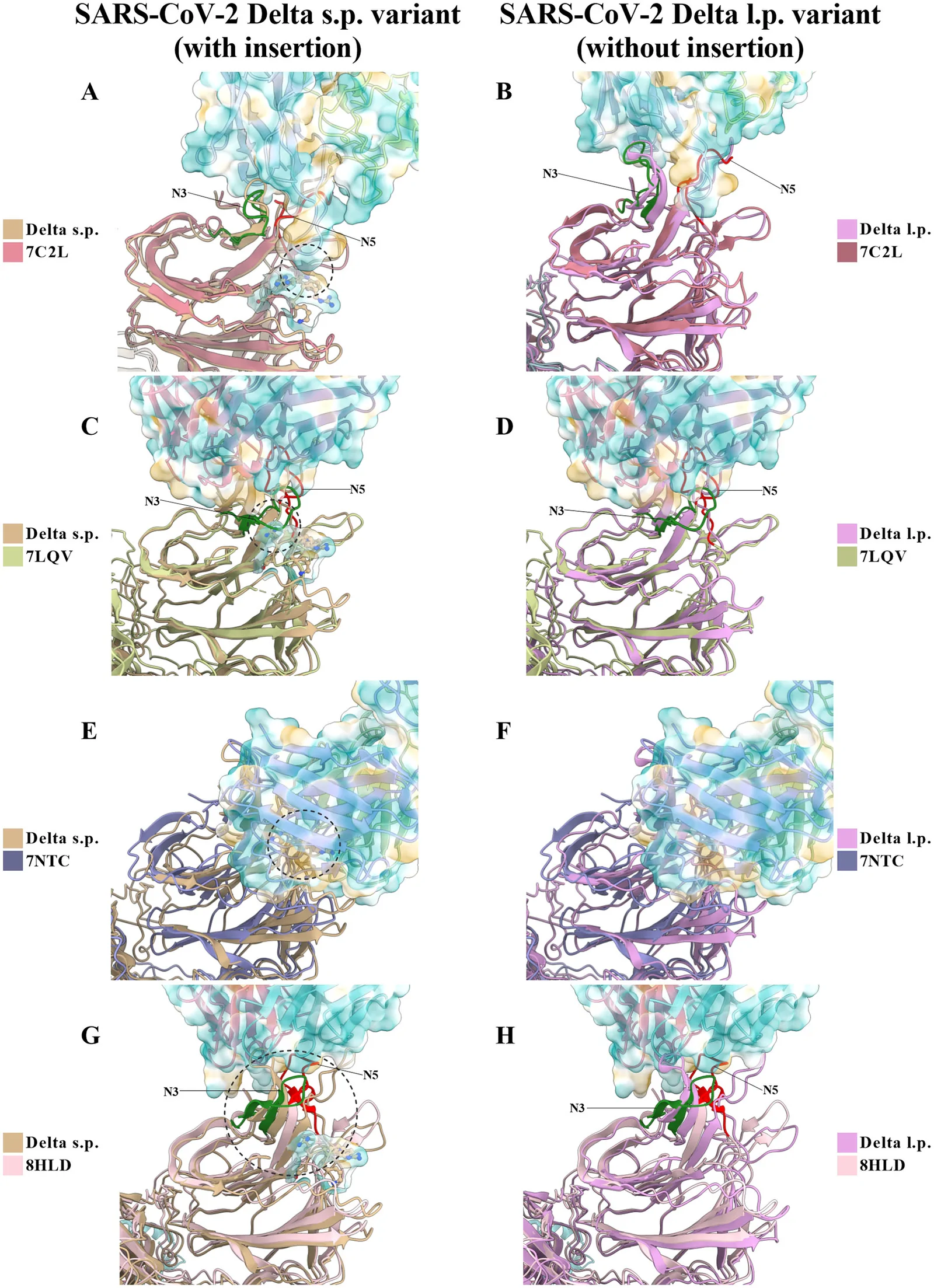
Spatial alignments of the N-terminal domain of the SARS-CoV-2 Delta s.p. (with insertion) and Delta l.p. (without insertion) Spike glycoprotein, constructed using the MatchMaker tool in ChimeraX: **(A)**, **(B)** with structure 7C2L (pink), containing antibody 4A8 (blue and light green chains, surface colored by hydrophobicity) **(C)**, **(D)** with 7LQV structure (yellow), containing antibody 4–8 (pink and purple chains, surface colored by hydrophobicity) **(E)**, **(F)** with structure 7NTC (purple), containing antibody P008_056 (purple and light green chains, surface colored by hydrophobicity) **(G)**, **(H)** with structure 8HLD (pink), containing antibody 26434 (dark pink and blue chains, surface colored by hydrophobicity). The amino acid residues of the insertion are shown as ball-and-stick models, with the surface visualized using hydrophobicity-based coloring. The N3 loop is marked in green, and the N5 loop is marked in red.

According to the literature, the most common antibody-binding epitope in the NTD is the “supersite”. It consists of regions N1–N5 (4–26 aa, 67–79 aa, 141–156 aa, 177–186 aa, 246–260 aa, respectively). Antibody 4A8, which belongs to the class of antibodies derived from the VH1–24 gene, binds to the N3 and N5 “supersite” loops in the 7C2L structure. The heavy chain of the antibody has three complementarity-determining regions: CDR1 (residues 25–32), CDR2 (residues 51–58) and CDR3 (residues 100–116). When the neutralizing antibody 4A8 interacts with the S protein NTD, the CDR3 region, which is inserted between loops N3 and N5, is located close to the insertion site we are studying, as shown in Figure 3A. Buried area (Figure 4A) and minimal distance (Figure 4B) analysis for the insertion conformation space revealed a wide range of values (953.73 ± 26.91 for models with antibody structures in reference and 805.28 ± 20.46 for models without), indicating possible interaction between the insertion loop and the antibody. The difference between the buried area ranges between the models built with and without presence of the antibody structure is due to the distinction in the conformations of the N5 loop (aa246–260), which is absent in the 7C2L structure. Clashes analysis performed with USCF ChimeraX (Supplementary Data 6, 7, Supplementary Figure S4) revealed Thr30, Pro106, Glu31, Leu32, Ala101, Gly104 and Asp107 as the most common antibody residues to overlap with the models generated in presence of the antibody structure, with clashes 30–32 and 101,104 being the result of the slight distinction in N3 loop conformation (NTD 141–156 aa), while Pro106 and Asp107 is the direct result of the overlap with the insertion residues. For the insertion conformations generated in absence of the antibody structure, Pro106, Phe109 and Thr105 became the most common overlaps, indicating that antibody loop 102–110 is causing changes in the generated insertion conformational space. The presence of the insertion may cause steric hindrance during the binding of the 4A8 antibody due to the mobility of the loops of the insertion and the heavy chain of the antibody. When the antibody interacts with the Delta l.p. variant of protein S (Figure 3B), such potential hindrances are absent.

**Figure 4:**
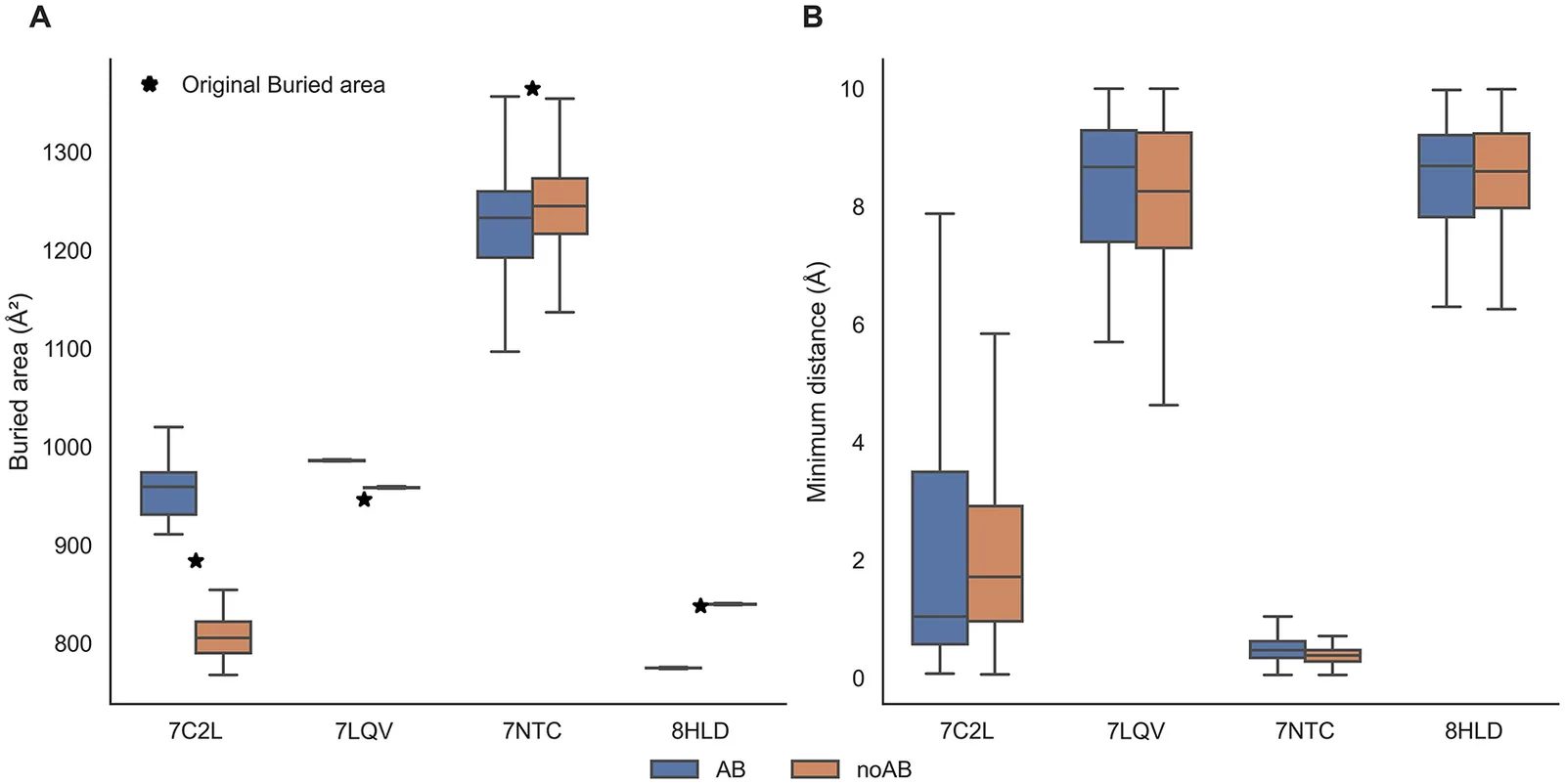
Boxplots of **(A)** buried area, Å^2^, and **(B)** minimum distance, Å, between the insertion atoms and antibody for models, built in presence (blue) and in absence (orange) of the antibody in the reference structure. For the buried area the value, derived from the reference structure is shown as a black star.

Another example of the possible interaction between the neutralizing antibody and the NTD is shown in Figure 3C. Antibody 4–8, belonging to the class of antibodies derived from the VH1–69 gene, (PDB ID 7LQV) interacts with the “supersite” loops N3 and N5, as does antibody 4A8, which belongs to a different class. However, the CDR3 of antibody 4–8 approaches orthogonally (the angle of approach is not longitudinal) to the region of the “supersite” loops, which differs from the interaction of antibody 4A8 with the NTD. Consequently, the mobile loops of the “supersite” adopt a different conformation upon interaction, as shown in Figures 3C, D. With this binding mode, where the N3 loop is located beneath the antibody's heavy chain and the N5 loop is located beneath the antibody's light chain, the presence of the insertion could hypothetically cause steric hindrance in the epitope region where the 4–8 antibody binds to the N3 loop, but as we see on the Figure 4A, there are no conformations, which directly contact with the antibody, thus changing the buried area value (986.12 ± 0.59 for models with antibody structures in reference and 958.72 ± 0.79 for models without). The limited amount of overlaps (Supplementary Data 8, 9, Supplementary Figure S5), appearing in the models are derived from the conformational change in the generated N3 loop, coming from the need to accommodate the insertion residues. This could hypothetically facilitate evasion of neutralizing antibodies targeting the N-terminal domain, which bind to the “supersite” orthogonally to its surface, but our computational approach does not allow to strictly confirm this. When studying the interaction with the variant without the insertion (Figure 3D), no steric hindrance may be observed.

The most telling case of the identified potential effect of the insertion on the interaction of S protein with neutralizing antibodies is that with those binding to the NTD “from the side” of the β-sandwich fold (Figure 3E). An example of this scenario is the interaction with the P008_056 antibody (PDB ID 7NTC). Upon binding of the P008_056 antibody, the NTD undergoes significant structural changes. Upon interaction with the antibody, loops N3 and N4, which form part of the “supersite”, bend significantly, and the antibody is located between them. The insertion is located within the structure between the described loops of the “supersite” and is thought to directly prevent antibody binding by altering the epitope and disrupting the complementarity of the binding interfaces. Even though the buried area values (Figure 4, Supplementary Data 4, 5) are lower, than the buried area in the reference structure due to the difference in the N3 loop conformation, we observe large dispersion of the values and very low minimum distances for both modeling regimes (buried aria 1224.76 ± 48.87 for models with antibody structures reference and 1244.42 ± 37.76 for models without). Clashes analysis (Supplementary Data 10, 11, Supplementary Figure S6) predictably reveals plenty of overlapping residues in both antibody chains, supporting the binding obstruction hypothesis.

We also examined cases where the identified insertion was predicted to have no effect on binding to antibodies targeting the NTD. To illustrate this, we chose to align the S protein models with the immune complex formed with antibody 26434 (PDB ID 8HLD). It binds to the N-terminal domain at the “supersite” but interacts with the NTD above the β-sandwich fold. With this binding mode, both the antibody and its associated heavy chains are distant from the insertion, so we do not expect any difference in antibody binding between the Delta s.p. (Figure 3G) and Delta l.p. (Figure 3H) models, which is supported by the absence of dispersion in the buried area values (774.85 ± 0.30 for models with antibody structures in reference and 839.80 ± 0.36 for models without) (Figure 4A) and large distance between insertion and antibody atoms (Figure 4B). The existing clashes (Supplementary Data 12, 13, Supplementary Figure S7) are caused by a slight difference in N3 loop conformation and are not expected to affect antibody binding.

## 4 Limitations

This study is based on comparative structural modeling and extensive conformational sampling of the NTD insertion, rather than on quantitative binding affinity measurements. While the generated ensemble enables assessment of whether the insertion can sterically affect antibody binding across multiple experimentally observed binding modes, it does not provide quantitative estimates of antibody affinity or binding free energy. Furthermore, our previous experimental validation was based on an ELISA method rather than on direct measurements of antibody binding, which precludes quantitative validation of computational binding energy predictions. Consequently, the structural models should be interpreted as illustrating plausible structural mechanisms underlying immune evasion rather than as a suitable data for quantitative predictors of antibody binding strength.

## 5 Discussion

Viruses with RNA-encoded genomes have the highest mutation rate among all biological agents due to the high number of errors in the functioning of RNA-dependent RNA polymerases and the absence of a repair system. They therefore constitute “quasi-species” with a vast diversity of offspring arising from errors during the replication of viral genomes (Bordería et al., 2015). Unlike other RNA viruses belonging to the quasi-species, SARS-CoV-2 has a region in its genome encoding a 3'-5' exonuclease (ExoN), which is thought to reduce the error rate of the replicase complex, as has been described for other coronaviruses, such as SARS-CoV and the mouse hepatitis virus. At present, it is difficult to assess the extent to which ExoN reduces the mutation rate, because SARS-CoV-2 replication gives rise to a large number of mutants, suggesting that the quasi-species theory may apply to SARS-CoV-2 (Domingo et al., 2023). At any given time, a quasi-species, including SARS-CoV-2, contains a large number of mutants that are theoretically capable of approaching a dynamic equilibrium. However, in nature, dynamic equilibrium does not exist, as the population is finite and constrained by environmental conditions. It is precisely this lack of population equilibrium that contributes to unpredictable changes in the frequency of the multitude of emerging and disappearing mutants, which increases the likelihood of viruses adapting, including to the host's immune response (Domingo and Perales, 2016). Among the mutations that arise, insertions and deletions have a greater impact on the viability of offspring than nucleotide substitutions. Their presence can lead to death, but they also drive viral evolution (Aguilar Rangel et al., 2023).

The functions of the NTD of SARS-CoV-2 remain poorly understood, but it has been suggested that the NTD may serve as a large and easily modifiable antigenic surface, enabling the virus to redirect the immune response away from the RBD and toward itself (Calvaresi et al., 2023). The literature describes antibodies against the NTD of the Spike glycoprotein with proven neutralizing activity, and their critical role in preventing infection has also been demonstrated. The binding epitopes of these antibodies are attractive targets for vaccine development (Suryadevara et al., 2025), which further confirms the importance of studying mutations, and even more so insertions, arising in the SARS-CoV-2 genome that encodes this region of the protein.

In our previous study, we not only identified two phenotypically homogeneous Delta variants, one of which contains a 12-nucleotide insertion in the NTD but also conducted a series of *in vivo* and *in vitro* experiments. In the *in vivo* experiment, which used the Syrian hamster as a model, we found that there was no significant difference in the course of the disease in the animals, including histological examination. Interestingly, animals infected with the Delta l.p. variant (without the insertion) lost more weight than those infected with the variant containing the insertion. However, *in vitro* experiments using the ELISA method, was shown that the Delta s.p. variant (with the insertion) binds less effectively to both polyclonal and monoclonal antibodies against the Spike glycoprotein (Ermolaeva et al., 2026).

Our work builds on previous research into the effect of insertions outside the “supersite” on the phenotypic properties of SARS-CoV-2. To this end, we investigated the potential impact of an insertion in the NTD of the Delta s.p. variant, obtained from a patient isolate, on binding to antibodies that specifically target various epitopes of the N-terminal domain that have been detected in patient sera. We analyzed cryo-EM structures of complexes between the Spike glycoprotein and antibodies to S protein NTD and selected four different binding modes, based on the potential impact of the insertion on the complex stability. Four represented structures were selected, which contained antibodies (4A8, 4–8, P008_056, 26434), which were obtained from patient sera and have been described in the literature (Chi et al., 2020; Cerutti et al., 2021; Rosa et al., 2021; Zhang et al., 2024).

Based on the spatial alignments performed for the N-terminal domain of the SARS-CoV-2 Delta s.p. (with insertion) and Delta l.p. (without insertion), we observe that the additional five residues-long loop in the domain's central region could hypothetically exert both direct and indirect effects on antibody binding to the NTD, including antibodies such as 4A8 (Klinakis et al., 2021), which bind to the “supersite” as well as to the side of the β-sandwich fold. This allows us to hypothesize that rapidly emerging mutations, particularly insertions, occurring in the region of interest—specifically outside the “supersite”—may potentially affect the binding by neutralizing antibodies targeted against the NTD of the Spike glycoprotein, with the exception of binding upstream of the β-sandwich fold (as is the case for the insertion we detected). The results we obtained could hypothetically apply not only to the Delta variant. If the same or a similar insertion were to occur in this region of the genome during replication in other coronavirus variants, it would likely have a similar effect, based on other previously described cases of mutations that arose independently in different coronavirus variants. For example, the N501Y mutation in Spike glycoprotein, which arose independently in variants such as Alpha, Beta, Gamma, and Omicron, led to increased affinity for the ACE2 receptor in all of these variants (Rajah et al., 2021; Hattab et al., 2024). Nevertheless there is no doubt that it is worth considering the possible influence of genetic background on the phenotypic expression of mutations (Moulana et al., 2026). Experimental verification is required to confirm the proposed hypothesis. In this regard, ongoing research into SARS-CoV-2 should focus more closely on rapidly emerging mutations in the region of the genome that encodes not only the NTD “supersite” but also other regions, as the NTD contains numerous immunogenic epitopes (Klinakis et al., 2021), and mutations occurring within it may help the virus evade the host immune response, whether generated through vaccination (Montgomerie et al., 2023) or following infection (Kubik et al., 2021).

## Data Availability

The datasets presented in this study can be found in online repositories. The names of the repository/repositories and accession number(s) can be found below: https://github.com/ErmolaevaAElena/2026_Ermolaeva_etal.
