# Correction: Insertion outside the supersite in the N-terminal domain of the SARS-CoV-2 spike glycoprotein may contribute to evasion of neutralizing antibodies

**DOI:** 10.3389/fmicb.2026.2018773

**Published:** 2026-10-22

**Authors:** Frontiers Production Office

**Affiliations:** Frontiers Media SA, Lausanne, Switzerland

**Keywords:** evasion of the immune response, glycoprotein spike, homology modeling, insertion, ntd, SARS-CoV-2

An incorrect number was provided for the *State Assignment*. The correct number is FNZG-2024-0005.

The original version of this article has been updated.

