# Supplementary material for: Insertion outside the supersite in the N-terminal domain of the SARS-CoV-2 spike glycoprotein may contribute to evasion of neutralizing antibodies
Source: Front Microbiol. 2026 Sep 18;17:1912073. doi: 10.3389/fmicb.2026.1912073 (PMC13631078; doi:10.3389/fmicb.2026.1912073)
Supplement: Supplementary file 1 [file Supplementary_file_1.docx]

Supplementary Material

**Supplementary Data**

**Supplementary Data 1.** Modeller template used for homology modelling of two SARS-CoV-2 Delta Spike glycoproteins (with and without an insertion) is available in a public GitHub repository: https://github.com/ErmolaevaAElena/2026_Ermolaeva_etal/tree/main/modeller%20template

**Supplementary Data 2.** Modeller script for insertion loop conformational sampling is available in a public GitHub repository: https://github.com/ErmolaevaAElena/2026_Ermolaeva_etal/tree/main/modeller%20template

**Supplementary Data 3.** Alignment of the two nucleotide sequences of the full-length SARS-CoV-2 genomes of the Delta s.p. and Delta l.p. variants: https://github.com/ErmolaevaAElena/2026_Ermolaeva_etal/tree/main/sequence%20alignments

**Supplementary Data 4.** Buried area, minimal distance between the insertion atoms and antibody atoms and DOPE scores for all insertion conformations, generated in presence of antibody structure:

https://github.com/ErmolaevaAElena/2026_Ermolaeva_etal/tree/main/Loop%20modeling%20results

**Supplementary Data 5.** Buried area, minimal distance between the insertion atoms and antibody atoms and DOPE scores for all insertion conformations, generated in absence of antibody structure:

https://github.com/ErmolaevaAElena/2026_Ermolaeva_etal/tree/main/Loop%20modeling%20results

**Supplementary Data 6.** Unfavorable spatial overlaps of the 1000 NTD models with insertion generated in presence of the antibody with structure 7C2L as reference.
https://github.com/ErmolaevaAElena/2026_Ermolaeva_etal/tree/main/Clashes%20analysis

**Supplementary Data 7.** Unfavorable spatial overlaps of the 1000 NTD models with insertion generated in absence of the antibody with structure 7C2L as reference.
https://github.com/ErmolaevaAElena/2026_Ermolaeva_etal/tree/main/Clashes%20analysis

**Supplementary Data 8.** Unfavorable spatial overlaps of the 1000 NTD models with insertion generated in presence of the antibody with structure 7LQV as reference.
https://github.com/ErmolaevaAElena/2026_Ermolaeva_etal/tree/main/Clashes%20analysis

**Supplementary Data 9.** Unfavorable spatial overlaps of the 1000 NTD models with insertion generated in absence of the antibody with structure 7LQV as reference.
https://github.com/ErmolaevaAElena/2026_Ermolaeva_etal/tree/main/Clashes%20analysis

**Supplementary Data 10.** Unfavorable spatial overlaps of the 1000 NTD models with insertion generated in presence of the antibody with structure 7NTC as reference.
https://github.com/ErmolaevaAElena/2026_Ermolaeva_etal/tree/main/Clashes%20analysis

**Supplementary Data 11.** Unfavorable spatial overlaps of the 1000 NTD models with insertion generated in absence of the antibody with structure 7NTC as reference.
https://github.com/ErmolaevaAElena/2026_Ermolaeva_etal/tree/main/Clashes%20analysis

**Supplementary Data 12.** Unfavorable spatial overlaps of the 1000 NTD models with insertion generated in presence of the antibody with structure 8HLD as reference.
https://github.com/ErmolaevaAElena/2026_Ermolaeva_etal/tree/main/Clashes%20analysis

**Supplementary Data 13.** Unfavorable spatial overlaps of the 1000 NTD models with insertion generated in absence of the antibody with structure 8HLD as reference.
https://github.com/ErmolaevaAElena/2026_Ermolaeva_etal/tree/main/Clashes%20analysis

**Supplementary Tables**

**Supplementary Table S1**. Models’ quality metrics

| Name | Description | Model name | DOPE | GA341 | ProSa z-score |
| --- | --- | --- | --- | --- | --- |
| SC2D | Delta s.p. full model | B99990001 | -397042.25 | 1.0 | -11.95 |
|  |  | B99990002 | -396498.84 | 1.0 | -11.87 |
|  |  | B99990003 | -395713.22 | 1.0 | -12.04 |
| SCDN | Delta s.p. NTD domain | B99990001 | -34167.32 | 1.0 | -5.68 |
|  |  | B99990002 | -34038.21 | 1.0 | -5.4 |
|  |  | B99990003 | -34038.73 | 1.0 | -5.47 |
| SDLP | Delta l.p. full model | B99990001 | -394827.22 | 1.0 | -11.91 |
|  |  | B99990002 | -395926.09 | 1.0 | -12 |
|  |  | B99990003 | -395820.34 | 1.0 | -12.03 |
| SLPN | Delta l.p. NTD domain | B99990001 | -33450.49 | 1.0 | -5.71 |
|  |  | B99990002 | -33703.91 | 1.0 | -5.49 |
|  |  | B99990003 | -33537.45 | 1.0 | -5.33 |

**Supplementary Figures**

**Supplementary Figure S1.** Alignment of amino acid sequences of Spike glycoprotein structures selected from the PDB for the construction of a homology-based Spike protein model (residues 54 –114). The region containing the described insertion is highlighted in red. The alignment was generated using MEGA-X 10.0.5. The complete alignment file is available in a public GitHub repository: (https://github.com/ErmolaevaAElena/2026_Ermolaeva_etal/tree/main/sequence%20alignments%20for%20modelling).

**Supplementary Figure S2.** Ramachandran plots for **(A)** the full-length Spike glycoprotein models and **(B)** Spike glycoprotein NTD models containing the insertion.

**Supplementary Figure S3.** Ramachandran plots for **(A)** the full-length Spike glycoprotein models and **(B)** Spike glycoprotein NTD models without the insertion.

**Supplementary Figure S4.** Unfavorable spatial overlaps of the 1000 NTD models with insertion generated in presence **(A)** and in absence **(B)** of the antibody with antibody residues (PDB ID 7C2L). Overlapping residues are shown as spheres. Supersite loops N3 and N5 are colored green and red respectively.

**Supplementary Figure S5.** Unfavorable spatial overlaps of the 1000 NTD models with insertion generated in presence **(A)** and in absence **(B)** of the antibody with antibody residues (PDB ID 7LQV). Overlapping residues are shown as spheres. Supersite loops N3 and N5 are colored green and red respectively.

**Supplementary Figure S6.** Unfavorable spatial overlaps of the 1000 NTD models with insertion generated in presence **(A)** and in absence **(B)** of the antibody with antibody residues (PDB ID 7NTC). Overlapping residues are shown as spheres. Supersite loops N3 and N5 are colored green and red respectively.

**Supplementary Figure S7.** Unfavorable spatial overlaps of the 1000 NTD models with insertion generated in presence **(A)** and in absence **(B)** of the antibody with antibody residues (PDB ID 8HLD). Overlapping residues are shown as spheres. Supersite loops N3 and N5 are colored green and red respectively.
